# Insanity

**Published:** 1876-05

**Authors:** 


### INSANITY.

One of the most affecting cases we ever knew was that of a woman. She loved most to talk about her children, of which she had six or seven. She was constantly engaged in the effort to repeat all their names, but never succeeded; one or another name would be left out, and then she would begin again.

The generally conceded truths, in reference to insanity, are:

More men become insane than women. The disease is more apt to terminate soon by death in men.

There is greater probability of recovery in women than in men; they bear confinement better. In very large cities, as New York and London, more men are cured than women.

In England, persons living in agricultural districts furnish a larger number of inmates for asylums than those living in manufacturing districts.

Married persons are more apt to recover than unmarried

More persons become insane who have not enough to do than of those who are full of business.

Of all who are insane, nearly one half become so from moral causes; such as anxiety, uncontrolled emotions, hugging sharp-pointed memories, &c.

Only nine persons out of a hundred are insane from hereditary causes.

The general truth seems to be, that of ten persons who are insane, five recover; and of these five, only two remain well during the remainder of life.

Of a hundred insane persons, thirty-one die within five years, and forty remain uncured. The ten years including the age of thirty, furnish very much the largest number of insane patients: showing that the buoyancy of youth and the maturity of riper years tend to avert insanity.

Those who are sent to the asylum within a year after their attack, get well in a large number of instances, while those who are kept at home for two or three years, until they become unbearable or dangerous, seldom recover, showing the immense value of scientific and experienced treatment. The two great practical lessons which stand boldly out from these statements, are: BE BUSY, if you would keep out of an asylum; but if attacked, send your friends there the moment the disease unmistakably manifests itself—a false delicacy or a mistaken affection often seals the doom for life, which might have—yes, would most probably have been averted.

